# Exclusive breast-feeding in the first six months: findings from a cross-sectional survey in Mulago hospital, Uganda

**DOI:** 10.4314/ahs.v22i2.62

**Published:** 2022-06

**Authors:** Michael E Otim, Elizabeth Kasirye Omagino, Amina Almarzouqi, Syed A Rahman, Augustine D Asante

**Affiliations:** 1 College of Health Sciences, University of Sharjah, PO Box 27272, Sharjah, UAE; 2 Institute of Health and Medical Research, University of Sharjah, PO Box 27272, Sharjah, UAE; 3 School of Public Health, Makerere University, PO Box 7062, Kampala Uganda; 4 School of Public Health and Community Medicine, University of New South Wales (UNSW) Sydney Australia

**Keywords:** Child Health, Mothers, Breastfeeding

## Abstract

**Background:**

Improving maternal and child health, one of the key UN Sustainable Development Goals (SDGs), is a major challenge in sub-Saharan Africa. Exclusive breast-feeding contributes significantly to child survival and development, but many mothers in Africa do not exclusively breastfeed their infants. This paper reports a study in Mulago hospital in Kampala. The study aims to identify factors influencing mothers' choices of infant feeding practices.

**Methods:**

Mixed methods were used. Respondents included 362 lactating mothers and health workers. Participants were who came for treatment were selected using simple random sampling. EpiInfor and SPSS were used for analysing the data and presented as descriptive study.

**Results:**

Results indicate that socio-demographic factors including age and education level influence mothers' ability and willingness to breastfeed exclusively for the first six months. Awareness about breast-feeding was mainly obtained from health centres, leaving mothers unable to attend these centres to miss out on vital information about exclusive breast-feeding. Around 43% of health workers were unaware of the country's Young and Infant Feeding Policy Guidelines.

**Conclusions:**

To increase the rate of exclusive breast-feeding in Uganda, it is important that community health is strengthened, and health workers are trained on national breast-feeding policies.

## Background

Improving maternal and child health in low and middle-income countries (LMICs) remains a global health priority. The United Nations (UN) Sustainability Development Goals No 3 (SDG 3) includes: “reducing the maternal mortality ratio to less than 70 per 100,000 live births by 2030 and ending preventable deaths of new-borns and children under 5 years of age, with all countries aiming to reduce neonatal mortality to at least as low as 12 per 1,000 live births and under-5 mortality to at least as low as 25 per 1,000 live births.”^1^. There has been considerable progress in reducing morbidity and mortality of mothers and children globally. The number of deaths among children under-five years old worldwide has declined from 93.1 per 1000 live births in 1990 to 39.1 per 1000 in 2017^2^. However, in sub-Saharan Africa progress towards targets for reducing child mortality has been slower than expected despite significant international efforts. Around half (2.8 million) of all the 5.4 million child deaths in 2017 occurred in sub-Saharan Africa 2. Children in sub-Saharan Africa are over 14 times more likely to die before the age of five than children in developed regions 3 .

Malnutrition is a major contributor to child morbidity and mortality, adding about 45% to the global statistics. It is estimated that sub-optimal infant feeding practices result in 1.4 million deaths and 10 percent of the disease burden in children younger than 5 years 4,5. The Sustainable Development Goals 2 and 3 focus on ending hunger and improving health and well-being 1. Breast-feeding is known to be a vital source of nutrition for children and plays a critical role in infant's healthy growth and development as well as reduction of infant morbidity and mortality and prevention of long-term chronic diseases^6^. Breast milk is known to have all the vitamins, minerals, enzymes and antibodies needed by a child to grow and thrive. This includes many immunological and anti-inflammatory properties that protect against many diseases for both mothers and newborns 7. Several studies in low- and middle-income countries (LMICs) have found that infants younger than 6 months who were not breastfed had 3•5-times (boys) and 4•1-times (girls) increases in mortality compared with those who received any breast milk, and that the protection decreased with age 8.

Exclusive breast-feeding (EBF) – defined by WHO as the practice of providing an infant with only breast-milk for the first six months of life, excluding solids or any other fluids except medicines, vitamins, and minerals – has been identified as having the single largest potential impact on child mortality of any preventive intervention^9^. Studies have found that breast-feeding exclusively for the first six months of life or longer has several important health benefits. For example, a German study found that infants exclusively breast fed for six months, compared with less than four months, had less gastroenteritis 10. A US cohort study showed that infants in US who were exclusively breast fed for more than six months had lower risk of pneumonia and recurrent otitis media than those breastfed for four to six months 11. The WHO recommends that all babies should be exclusively breast fed from birth until six months of age, and that mothers should be counselled and provided support for exclusive breast-feeding at each postnatal contact 12.

Despite the immense benefits of EBF, less than 40 percent of infants under six months of age are exclusively breastfed worldwide 13. In sub-Saharan Africa, where malnutrition in children is more prevalent, just about 37% of infants under six months are exclusively breast-fed 14,15. This is despite breast-feeding being a widespread practice on the continent. In countries such as Chad and Sierra Leone, the rate of EBF is below 5 percent 16,17 as observed that in most of sub-Saharan Africa, the increased levels of undernutrition are strongly associated with the age of the child. Uganda is no exception.

## Child health and exclusive breast-feeding in Uganda

Uganda, with a population of 32 million, has made considerable progress in improving child health. Under-five mortality rate has fallen from 181.2 per 1000 live births in 1990 to 49 per 1000 in 2017. Infant mortality rate, which measures deaths among children less than 1 year of age, has dropped from 97 per 1000 live births to 54 over the same period^18^,^19^. The slow progress has been in part because of poor nutrition and suboptimal feeding, such as the low rate of EBF.

Malnutrition has significantly contributed to the slow progress in improving child health in Uganda. The Ugandan Child Survival Strategy estimates that malnutrition directly and indirectly contributes up to 60 percent of child mortality^20^. At the national level, the country produces sufficient food to meet the needs of the population. However, a high number of Ugandans are unable to access recommended calories due to a range of factors including uneven distribution of food and inequalities in wealth^21^. There are significant regional variations in the prevalence of malnutrition among children under-five years of age. For example, stunting and wasting are highest in Karamoja while underweight is highest in East and Central Ugandan regions^22^.

Furthermore, suboptimal breast-feeding of infants and children is a major contributor to childhood malnutrition in Uganda. Optimal breast-feeding is defined as exclusive breast-feeding for the first six months and continued breast-feeding with adequate complementary foods from six months to two years or beyond 21. Breast-feeding is nearly a universal practice in Uganda with about 98% of children having been breastfed at some point in time. The government recommends exclusive breast-feeding for all infants whose mothers are HIV negative or do not know their HIV status. HIV positive mothers are advised to exclusively breastfeed their infants for 6 months unless replacement feeding meets the criteria of acceptable, feasible, affordable, sustainable and safe (AFASS)^23^.

The widespread practice of breast-feeding in Uganda, as in many other African countries, does not translate into high EBF rates. Many children in Uganda are not exclusively breastfed. It is estimated that only about 10 percent of children under the age of six months are exclusively breastfed 21, although a study in eastern Uganda reports a rate of about 20 percent 24. Many infants under 6 months in Uganda are introduced to fluids other than breast milk. About 16% of infants are given fluids other than breast milk or foods by one month of age with the proportion rising to about 65 percent between 4–5 months 22. To improve child health in Uganda, it is paramount that the reasons for the low rate of exclusive breast-feeding of infants are understood and addressed by policy decisions to improve child survival. We report a study that investigates breast-feeding practices of lactating mothers of infants under six months of age accessing health care at Mulago hospital in Kampala. The study aims to identify factors influencing mothers' choices of infant feeding practices and recommends options for improvement.

## Methods

### Design, study site and population

A cross-sectional study using a mixed of quantitative and qualitative methods was undertaken. The study was carried out at Mulago hospital in Kampala, the capital city. Mulago is a national referral and teaching hospital with a bed capacity of 1,500. Mulago hospital Assessment Centre (MAC) has a number of outpatient clinics including the MAC paediatric outpatient clinic for children below 12 years of age. The study population included 332 lactating mothers with children of less than one year old attending the MAC pediatric outpatient clinic. A simple random sampling procedure was followed in selecting the participants (the mothers). The health workers, majority of whom were nurses, were purposefully selected to participate in key informants.

### Data collection

Both quantitative and qualitative data between October and November 2012. First, a structured questionnaire was administered to the 332-lactating mother by one of the authors and two trained research assistants. This included family background such as living conditions and mother's education level, occupation, and breast-feeding practices and knowledge about exclusive breast-feeding, including sources of information on EBF among other things. There were both closed and open-ended questions and respondents selected answers that best described their situation and also permitted them to provide greater depth of response and express personal opinions about the subject. Respondents who could read and write and wanted to complete the questionnaire themselves could do so. For those who were not able to do so, the questionnaires were read to them by one of the researchers. All questionnaires were pre-tested to ensure that all questions worked well, and modifications were made where necessary.

Second, key informant interviews (KIIs) were held with the 30 purposefully selected health workers to explore their views about exclusive breast-feeding and the policies around breast-feeding as well as their experiences in educating mothers about breast-feeding. The KIIs were guided by a pre-designed interview schedule and were conducted by only the first author. Finally, two focus group discussions (FDGs) were held with selected mothers to explore EBF in more detail and cultural behaviours relating to breast-feeding. Each FDG had nine participants.

### Ethics Approval

Ethics approval for the study was obtained from Uganda Christian University Research Board and from the Uganda National Council of Science and Technology.

### Data analysis

The quantitative data were organised, coded and entered using EPI data 3.1. Descriptive analysis of the quantitative data was undertaken using SPSS version 16. Means and frequencies are presented along with 95% confidence intervals. The qualitative data obtained from open ended questions, the KIIs and FDGs were analysed using thematic content analysis. Short summaries of themes were compiled and coded. Where possible the qualitative data were triangulated with the quantitative data to establish validity.

## Results

### Socio-demographic characteristics of respondents

Ninety-two percent of the 332 breast-feeding mothers were between the ages of 18 – 39 years old and about 74 percent were married (Table 1). Nearly 41 percent of the mothers surveyed were engaged in some form of formal work, and about one third were full-time housewives. Sixty percent of these respondents had attended at least a secondary school level of education, and 20 percent were HIV positive. Of the 30 health workers who served as key informants, 80 percent were females and around 70 percent were between the ages of 20 – 30 years.

**Table 1:** Socio-demographic characteristics of the study participants and relationship with exclusive breast-feeding

	Characteristics of study population		Exclusive breast-feeding practices	
Mothers surveyed	Frequency (n)	Percentage (%)	Yes *n (%)*	No *n (%)*
**Age group**				
*14 – 17*	3	0.9	-0 (0.0%)	3 (100%)
*18 – 29*	209	63.0	128 (61.2%)	81 (38.8%)
*30 – 39*	99	29.8	74 (74.7%)	25 (25.3%)
*40 – 49*	18	5.4	15 (83.3%)	3 (16.7%)
*50 – 59*	3	0.9	3 (100%)	0 (0.0%)
**Occupation**				
*House wife*	119	35.8	98 (82.3%)	21 (17.8%)
*Civil Servant*	40	12.0	16 (40.0%)	24 (60.0%)
*Business Woman*	96	28.9	53 (55.2%)	43 (44.8%)
*Others*	77	23.3	51 (69.9%)	22 (30.1%)
**Education Level**				
*Tertiary*	53	16.0	23 (43.4%)	30 (56.6%)
*Secondary*	146	44.0	88 (60.3%)	58 (39.7%)
*Primary*	119	35.8	93 (78.1%)	26 (21.8%)
*None*	14	4.2	14 (100%)	0 (0.0%)
**Marital Status**				
*Married*	244	73.5	172 (70.5%)	72 (29.5%)
*Single*	62	18.7	29 (47.5%)	32 (52.5%)
*Divorced*	11	3.3	9 (81.8%)	2 (18.2%)
*Cohabiting*	9	2.7	4 (44.4%)	5(55.6%)
*Widowed*	6	1.8	6 (100%)	0 (0.0%)
**Sero Status**				
*Positive*	66	19.9	47 (71.2%)	19 (28.8%)
*Negative*	247	74.4	153 (61.9%)	94 (38.1%)
*Unknown*	19	5.7	16 (84.2%)	3 (15.8%)

### Exclusive breast-feeding and socio-demographic characteristics of respondents

We found that the age and level of education influenced the mothers' willingness and ability to breastfeed exclusively for the first six months (Table 1). Mothers, who have the basic knowledge about breast-feeding, however far they lived from the health workers, were likely to breast feed exclusively. Results indicate that exclusive breast-feeding improved as the age of the mother increased (Table 1).

The higher the educational attainments, the less likely the mothers were to exclusively breastfeed. For example, all mothers without formal education reported to be exclusively breast-feeding their babies (100 percent), and those with tertiary education were the least likely (43.4 percent). Being HIV positive did affect the mother's choice of feeding her infant. 71 percent of the mothers with positive sero-status were found to be breast-feeding exclusively for the first six months, compared to 62 percent of those with negative sero-status (Table 1).

### The infant feeding practices of mothers of infants less than six months accessing care in Mulago hospital

Results revealed that about 72 percent of the mothers were willing, and the same proportion had the intention to exclusively breastfeed for 6 months. However, due to inadequate breast milk, and health reasons, only about 67 percent of mothers breastfed exclusively (Table 2).

**Table 2:** Factors affecting the choice of infant feeding practice

Factors hindering mothers from exclusive breast- feeding for 6 months			Showing age at which infants are given additional feeds		
Factors	Frequency	Percent	Months	Frequency	Percent (%)
Low breast milk	14	4.2	1 month	76	22.9
Work/business pressure	38	11.5	2 months	24	7.2
Stopped (Health issues)	3	0.9	3 months	19	5.7
Poor feeding	2	0.6	4 months	13	3.9
Habit	1	0.3	5 months	6	1.8
Others	21	6.3	6 months	121	36.4
Unknown	253	76.2	None	73	22.0
Total	**332**	**100**	Total	**332**	**100**

We found that about 36 percent of the infants had been introduced to additional feeds by the age of six months (Table 2). Mothers were aware of the pros and cons of exclusive breast-feeding, and the main source of such awareness was received mainly from health centre workers. Results indicate that the majority of mothers (96 percent) were aware of the effects of exclusive breast-feeding to their children (Table 4). These included ill health (35 percent), malnutrition, poor/retarded growth, intellectual disability, depression and other disorders.

**Table 4:** Assessing knowledge, attitudes and beliefs of the mothers on infant feeding and exclusive breast-feeding

Reasons for continued exclusive breastfeeding			Recommended period for breast-feeding before other feeds		
Reasons/Factors	Frequency	Percent	Period/Factors	Frequency	Percent
Best food for infants and babies	27	8.1	Less than 6 months	2	0.6
Good Health	48	14.5	6 months	309	93.1
Enables the baby to grow well	38	11.5	More than 6 months	8	2.4
Contains all the nutrients the baby needs	8	2.4	Do not know	13	3.9
Tradition and responsibility	17	5.1	**Total**	**332**	**100**
Others	189	56.9			
Unknown	5	1.5			
**Total**	**332**	**100**			
					
Prevents HIV mother to child transmissions	1				
Breast-feeding is easy and cheap to administer	3	10			
Creates love between the mother and the baby	1	3.3			
Reduces malnutrition	1	3.3			
Prevents diarrhoea	2	6.7			
Mixed feeding increases chances of mother to child transmission of HIV	1	3.3			

### Assessing the knowledge, attitude and belief of the mothers on infant feeding and exclusive breast-feeding

The main source of health information to mothers regarding recommended infant feeding guidelines was from health workers (75.3 percent). Other sources included the mass media (radio and TV) and friends (Table 3). Community health workers accounted for only 2.4 percent. These findings were confirmed by the focus group discussions.

**Table 3:** Source of Information on breast-feeding given to mothers

Information Source	Frequency	Percent
Radio	53	16.0
Friend	14	4.2
Health workers	250	75.3
Community resource persons	8	2.4
Other sources	5	1.5
Unknown	2	0.6
Total	332	100

Mothers of infants younger than 6 months revealed that good health for the baby, breast milk enables the baby to grow well (11.5 percent), best food for infants (8.1%), contains all nutrients the baby needs (2.4 percent), and tradition (5.1 percent) encouraged them to feed their babies (Table 4). These results were confirmed by the focus group discussion.

Mo thers' knowledge on the recommended period for exclusive breast-feeding for the first 6 months was also elicited (Table 4). Ninety three percent of the respondents indicated their awareness.

### Assessing the knowledge, attitude and beliefs of the health workers on exclusive breast-feeding

To confirm the above results, on recommended period for exclusive breast-feeding, we held focus discussion groups. These groups revealed similar levels of awareness. It also revealed that 43.3 percent of health workers had no knowledge about the Young and Infant Feeding Policy Guidelines (Table 5).

**Table 5:** Assessing knowledge, attitudes and beliefs of the Health Workers on exclusive breast-feeding

Key informants' knowledge about exclusive breast-feeding			Key informants Knowledge about benefits of general breast-feeding		
	Freq (n)	Percent		Freq (n)	Percent
Exclusively breast-feed for the first 6 months	23	76.7	Breastfeed for the first 6 months	7	23.4
Breast feed 8 times in 24 hours	3	10	Breast-feeding gives the right balanced diet	4	13.4
Creates bond between the baby and the mother	6	20	None	13	43.3
Good growth and development	5	16.7	Breast-feeding contains vitamins and proteins the babies need for their growth	1	3.3
Provides and boost immune system	3	10	Breast-feeding is compulsory	1	3.3
Contains the nutrients the baby needs	3	10	Positive mothers should mix breastfeeding with other feeds	1	3.3
Breast-feeding prevents diseases	2	6.7	Mothers should be advised to breastfeed regularly	2	6.7
Means of family planning	2	6.7	Breastfeed for 2 years	1	3.3

Among the key informants, 78 percent were aware that infants are supposed to breastfeed exclusively for the first six months of their life (Table 5). Unfortunately, only 16.7 percent knew that breast-feeding leads to good growth and development of the child whereas the rest revealed that it provides and boosts the infant's immunity. When asked about infant and baby feeding policy guidelines, health workers lacked such knowledge or any information on exclusive breast-feeding. Only 23.4 percent had some knowledge about it.

One health worker stated:

“*Mothers can breastfeed their children as long as they want. For HIV positive mothers, exclusive breast-feeding is recommended for 6 months or longer provided both the mother and child are on Anti-Retroviral Treatment - ART*”.

Another worker added:

“*The child is introduced to any other foods that are soft, with blunt flavours i.e. not too sweet, not too salty such that the child grows to appreciate different tastes. Ensure that the food introduced is a balanced diet*”.

From the FGDs, majority of the participants revealed that they had knowledge on the recommended period for exclusive breast-feeding and knowledge on recommended period for exclusive breast-feeding, and the benefits of breast-feeding. From the FGDs, majority of the participants revealed that they had knowledge on the recommended period for exclusive breast-feeding and knowledge on recommended period for exclusive breast-feeding, and the benefits of breast-feeding. These groups were interviewed under three themes: breast milk content; benefits of breastfeeding and the Infant and Young Child policy guideline recommended period of exclusive breastfeeding. In response, majority had knowledge on the recommended period for exclusive breastfeeding and knowledge on recommended period for exclusive breastfeeding, and the benefits of breastfeeding.

## Discussion

The primary aim of the study was to identify factors associated with exclusive breast-feeding among mothers who accessed infant and young child care in Mulago Hospital. The information generated would assist in improving the existing infant feeding Policy Guidelines and re-strategize implementation of Young and Infant Feeding Policy Guidelines through effective public education.

The results indicated that majority of the mothers breastfed their babies. The higher the educational attainments, the less likely the mothers were to exclusively breast-feed. For example, all the mothers without formal education reported to be exclusively breast-feeding their babies, and those with tertiary education were the least likely to exclusively breast-feed. Socio-demographic characteristics such as age, affects general breast-feeding and exclusive breast-feeding. These findings are consistent with findings reported in other studies; that the ability of mothers to breast-feed correctly seems to be a learned experience^25^.

In general, factors that affect the mothers' ability to exclusively breast-feed can be loosely grouped into personal and institutional issues. Personal factors such as occupation or levels of education may affect a mother's breast-feeding practices. This is consistent with findings in other studies suggesting that mothers who are not engaged in formal employment had more time to breastfeed as compared to those that are formerly employed^26^. Limitations on breast-feeding at workplace may be a contributory factor. Political will has potential to improve breast-feeding such as the nursery opened in the Uganda Parliament to allow members of parliament to feed their babies. Therefore, it is important for the work environment to encourage breast-feeding by providing facilities for lactating mothers, example, providing breast-feeding corners that mothers can conveniently use during work hours. Furthermore, government should ensure that mothers get paid parental leave for at least the first six months.

This could be attributed to the fact that the mothers in this study have access to health facilities where they attended maternity care. However, mothers in the rest of Uganda rural settings do not often have access to health centres or trained health workers. Thus, their knowledge and understanding of exclusive breast-feeding may be minimal. For example, awareness of the pros and cons of exclusive breast-feeding was received mainly from health centre workers, yet 43.3 percent of health workers had no knowledge about the Young and Infant Feeding Policy Guidelines^20^.

## Conclusion

The rate of exclusive breast-feeding in Uganda is slightly higher than in other developing countries. Over the last five years, the prevalence has slightly increased by about 2 percent. Socio demographic factors appear to have considerable influence on the mothers' ability and willingness to exclusively breastfeed. These factors include age, level of formal education, employment type, and marital status. Most mothers wean their babies after six months and this is in tandem with the WHO and Uganda's Ministry of Health guidelines on exclusive breast-feeding. To increase the rate of exclusive breast-feeding in Uganda, it is important that community health is strengthened, and health workers are provided training on national policies around breast-feeding and child nutrition so as to educate mothers on weaning and nutrition.

## Data Availability

Available upon request subject to ethical restriction.

## Abbreviations

HIV: Human Immunodeficiency Virus
EBF: Exclusive Breast Feeding
MAC: Mulago hospital Assessment Centre
WHO: World Health Organisation
FDG: Focus Group Discussions
SDG: Sustainable Development Goals
LMIC: Low- and Middle- Income Countries
KII: Key Informant Interviews
MDG: Millennium Development Goals
SPSS: Statistical Package for the Social Sciences
OECD: Organisation for Economic Cooperation and Development
